# The comparison of different antiviral therapies on the prognosis of hepatitis B virus-related hepatocellular carcinoma after curative treatments: A network meta-analysis

**DOI:** 10.1097/MD.0000000000020877

**Published:** 2020-08-14

**Authors:** Zijing Xia, Linye He, Li Xiong, Tianfu Wen

**Affiliations:** a Department of Rheumatology and Immunology; b Department of Thyroid & Parathyroid Surgery, West China Hospital, Sichuan University, No. 37 Guo Xue Xiang, Chengdu, Sichuan Province; c Department of Pharmacy, The First Affiliated Hospital of Chongqing Medical University, Chongqing 400016; d Department of Liver Surgery & Liver Transplantation Center, West China Hospital, Sichuan University, No. 37 Guo Xue Xiang, Chengdu, Sichuan Province, PR China.

**Keywords:** antiviral therapy, hepatitis B virus, hepatocellular carcinoma, network meta-analysis, nucleos(t)ide analogues

## Abstract

**Objective:**

The purpose of this study was to evaluate the efficacy of different nucleos(t)ide analogues in the prognosis of HBV-related hepatocellular carcinoma (HCC) patients after curative treatment by network meta-analysis.

**Methods:**

Literature retrieval was conducted in globally recognized databases, namely, PubMed, EMBASE, Cochrane Library databases, and Science Citation Index Expanded, to address relative studies investigating nucleot(s)ide analogues for HBV-related HCC patients after curative resection. Relative parametric data, including 1-, 3-, and 5-year overall survival rate and 1-, 3-, and 5-year recurrence-free survival rate were quantitatively pooled and estimated. The inconsistency factor, the cumulative ranking curve, and the publication bias were evaluated.

**Results:**

Fourteen observational studies of 2481 adults performed between 2000 and 2019 were eligible. In terms of overall survival, ADV (Adefovir dipivoxil) (Odds ratio (OR): 2.35, 95% confidence interval (CI): 1.17–4.73), Lamivudine (OR: 2.08, 95% CI: 1.78–5.58), and Entecavir (OR: 2.14, 95% CI: 1.59–2.88) were found to be more beneficial than control group while ADV has the highest probability of having the most efficacious treatment (SCURA values 66.3) for 5-year overall survival. In late recurrence-free survival, ADV (OR = 1.88, 95% CI: 1.77–4.60), Entecavir (OR = 1.96, 95% CI: 1.36–2.55), and Lamivudine (OR = 1.73, 95% CI: 1.06–2.82) all had better significant prognosis than patients without antiviral therapy postoperatively and patients with ADV as postoperative antiviral therapy has significantly recurrence-free survival benefit at 5-year follow-up compared to those undertaking Entecavir (OR = 1.96, 95% CI: 1.52–7.38) and Lamivudine (OR = 1.39, 95% CI: 1.09–3.01). Moreover, the application of ADV possessed the highest possibility of having the best clinical effects on 1- (surface under the cumulative ranking probabilities (SUCRA), 64.7), 3- (SUCRA, 64.7), and 5-year (SUCRA, 70.4) recurrence survival rate for HBV-related HCC patients.

**Conclusions:**

Patients with postoperative nucleos(t)ide analogues antiviral therapy had better survival benefit than those without antiviral therapy for HBV-related HCC patients after curative treatment. Additionally, nucleotide analogues like ADV and Tenofovir disoproxil fumarate has better impact on early and late recurrence-free survival of patients after curative treatment than those undertaking nucleoside analogues.

## Abstract 2

Supplemental Digital Content is available in the text

## 1 Introduction

In Asia, hepatocellular carcinoma (HCC) commonly occurred in the underlying hepatitis B virus (HBV)-related liver disease.^[1]^ Curative therapies like liver transplantation, hepatectomy, and radiofrequency ablation could improve the prognosis of HCC patients. With the improvement in surgical techniques and advancement in preoperative assessment, the 5-year survival rates after curative therapy has reached 50%.^[2]^ However, tumor recurrence after curative therapy remains high with a 5-year recurrence rate >70%.^[3,4]^ To date, no universally effective adjuvant treatment has been available to prevent HCC recurrence.

Chronic HBV infection is the main cause of HCC in Asia. The risk for HCC development is increased for patient with HBV infection.^[5,6]^ Recent studies also showed that tumor recurrence after curative treatment of HCC was increased with the level of HBV-DNA and alanine aminotransferase (ALT).^[2,7]^ Studies of large cohorts from China Hong Kong, China Taiwan, and Japan have confirmed that concomitant antiviral therapy with curative treatment reduced the recurrence of HCC.^[8–22]^ However, there was no consensus about which kind of oral antiviral treatment was the best option in the prevention of HBV-related HCC recurrence after curative treatment. The aim of this network meta-analysis was to sum up the current evidence about the efficacy of different nucleos(t)ide analogues on the prognosis of HBV-related HCC after curative treatment.

## 2 Methods

Our study was approved by the local institutional review board and was conducted in compliance with the Health Insurance Portability and Accountability Act of 1996. Our study protocol was received and approved by the Medical Ethics Committee of Sichuan University. Written informed consent was obtained from all participants.

### 2.1 Eligibility criteria for this review

This analysis was performed in accordance with the PRISMA (Preferred Reporting Items for Systematic Review and Meta-Analyses) statement.^[23]^ Studies were included if they fulfilled the following inclusion criteria:

(1) Randomized trials or cohort design.
(2) HBV infection treated by nucleotide/nucleoside analogs after curative therapy.
(3) HCC treated by curative surgical resection or ablation therapy.
(4) Complete follow-up data about recurrence-free survival (RFS) or overall survival (OS).
(5) Studies including nucleotide/nucleoside analogs therapy compared with placebo or no treatment after curative therapy of HCC.

Exclusion criteria were:

(1) Studies involving in hepatitis C and/or D-related HCC.
(2) Patients received liver transplantation, interferon-based antiviral therapy, or pallitive treatment.
(3) Patients received antiviral treatment preoperatively.

Patients in the study received either Entecavir (ETV) tablets (RunZhong, CHIATAI TIANQING) 0.5 mg/day, Tenofovir disoproxil fumarate (TDF) tablets (Viread, Aspen Port Elizabeth) 300 mg/day, Adefovir dipivoxil (ADV) tablets (Hepsera, GlaxoSmithKline) 10 mg/day, Lamivudine (LAM) tablets (Heptodin, Ameresco) 100 mg/day, Telbivudine (LdT) tablets (Subivine, Nuohua), 600 mg/day orally or did not take antiviral therapy at all. Patients resistant to entecavir or lamivudine were recommended to add adefovir or switch to tenofovir.

### 2.2 Data sources and search strategy

**Figure 1:**
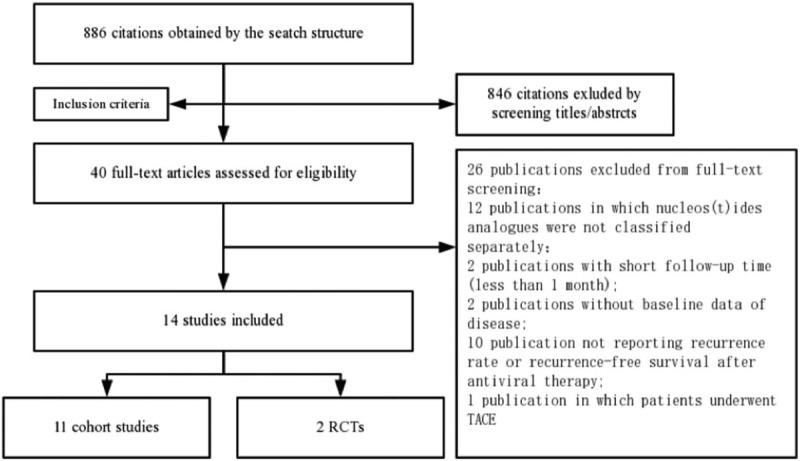
Flow chart of search and studies selection process. RCT, randomized controlled trial; TACE, transarterial chemoembolization.

We conducted a systematic search using the following electronic data-bases from inception to February 2019: PubMed, EMBASE, Cochrane Library databases, and Science Citation Index Expanded without language restriction. The search strategy was based on MeSH terms, combined with free text words. The detailed strategies are given in Figure 1. Reference lists of all identified papers (included studies and relevant reviews) were checked for additional studies suitable for inclusion. Our primary aim was to identify different effect of nucleos(t)ide analogues as antiviral therapy to postoperative survival of HBV-related HCC patients after radical hepatectomy.

### 2.3 Data collection and assessment of bias

Two investigators independently reviewed all the titles, abstracts, and manuscripts identified to determine if each study was eligible for inclusion in the meta-analysis, as recommended by the Cochrane Handbook for RCTs^[24]^ and the Newcastle–Ottawa Scale (NOS) for observational studies.^[25]^ Disagreements about eligibility were resolved by consensus with a third reviewer. A structured search using keywords (“hepatitis B virus,” “chronic hepatitis B” or “hepatitis B” or “HBV” or “CHB”), (“hepatocellular carcinoma” or “liver cancer” or “primary liver carcinoma”), (“antiviral therapy” or “Nucleotide” or “Nucleoside” or “lamivudine” or “adefovir” or “entecavir” or “tenofovir” or “telbivudine”), and (“randomized trial” or “random- ized” or “randomized controlled trial (RCT)”) or (“retrospective” or “prospective” or “cohort”) was performed. Data were extracted from each study with a predesigned review form as follows: study characteristics (publication year, publication type, journal, country, study design, disease population, inclusion criteria, and exclusion criteria); patients’ baseline characteristics (age, gender, weight); HBV-related disease information (marrow function, liver function, viral information); tumor features (tumor size, tumor number, tumor encapsulation, satellite nodule, vascular invasion, tumor differentiation, tumor stages, and alpha fetoprotein (AFP); treatment information (follow-up duration, intervention drug, drug dose, tumor treatment type); and clinical outcomes (1-, 3-, and 5-year RFS or OS).

### 2.4 Statistical analysis

We performed a pairwise meta-analysis using STATA 14.0 software (Stata Corporation, College Station, TX). 1-, 3-, and 5-year RFS rate and 1-, 3-, and 5-years OS were defined as the endpoint. Odds ratio (OR) with 95% confidence intervals (CIs) was calculated using the random-effects model or fixed-effects model for investigating treatment effects.^[26,27]^*Z* test was conducted to assess the significance of overall effect size. *P* value of less than .05 was considered statistically significant.

A network plot was produced to represent the overall information of the trials included in the analysis. Nodes size represents the number of trials for each treatment and lines thickness represents the number of available direct comparisons.^[28]^ The contribution of each direct comparison to each network estimate was calculated according to the variance of the direct treatment effect and the network structure, later summarized in a contribution plot.^[29]^

After constructing a heterogeneity matrix, the frequentist method was applied to the fitted meta-regression model. The model covariates as the basic parameters and assumed that heterogeneity is independent of the comparison between effect sizes from multi-arm studies.^[30,31]^ A forest plot of the estimated summary effects, along with confidence intervals and corresponding predictive intervals (PrI) for all comparisons, summarizes the relative mean effects, the impact of heterogeneity, and predictions on each comparison in 1 plot.^[32]^

To rank the treatments, we used the surface under the cumulative ranking probabilities (SUCRA); a SUCRA value of 100% is assigned to the best treatment and 0% for the worst treatment.^[33]^ A comparison adjusted funnel plot was used to assess the presence of small-study effect.^[34]^ Egger's test was used to assess the symmetry of the funnel plot.^[35]^

To account for both the markedly effective rate and neurological deficits, we used multivariate methods to determine the dependency between outcomes. Clustering methods and 2-dimensional plots were used to produce clusters of treatments.^[36]^ Using the clusterank command, clustered ranking plots can be obtained using the STATA program. The markedly effective rate and neurological deficits became the data variable containing the SUCRA scores for all treatments in this network. The different colors correspond to the estimated clusters and were utilized for grouping the treatments according to their similarity for the outcomes.

## 3 Results

### 3.1 Characteristics of the studies included

A total of 886 publications were identified in the initial search and 846 records were excluded based on the screening of titles, abstracts, or duplicate articles (Fig. 1). Full-text articles were retrieved for the 40 remaining publications and assessed for eligibility for inclusion. Of these 40 publications, 26 were excluded (12 publications in which nucleoside/nucleotide analogues were not classified separately, 2 publications with antiviral therapies before curative treatment, 2 publications with short follow-up time (less than 1 month), 10 publications not reporting recurrence rate or recurrence-free survival after antiviral therapy, 1 publication in which patients underwent transarterial chemoembolization (TACE)). Therefore, 14 remaining studies qualified for study inclusion. The 14 selected studies were published between 2000 and 2019 and involved a total of 2481 patients.^[9–11,14,18,21,22,37–43]^ The longest median follow-up duration was more than 120 months and the shortest median follow-up duration was 60 months. Study characteristics are summarized in Table 1. These studies were performed in 3 different countries (5 in Japan,^[9,40–43]^ 8 in China mainland,^[10,11,14,18,21,22,37,38]^ and 1 in the Korea^[39]^); all studies were published in full text. The type of study design included 1 case–control studies,^[40]^ 11 retrospective observational studies,^[9–11,14,22,37–39,41–43]^ and 2 RCTs.^[18,21]^

**Table 1:**
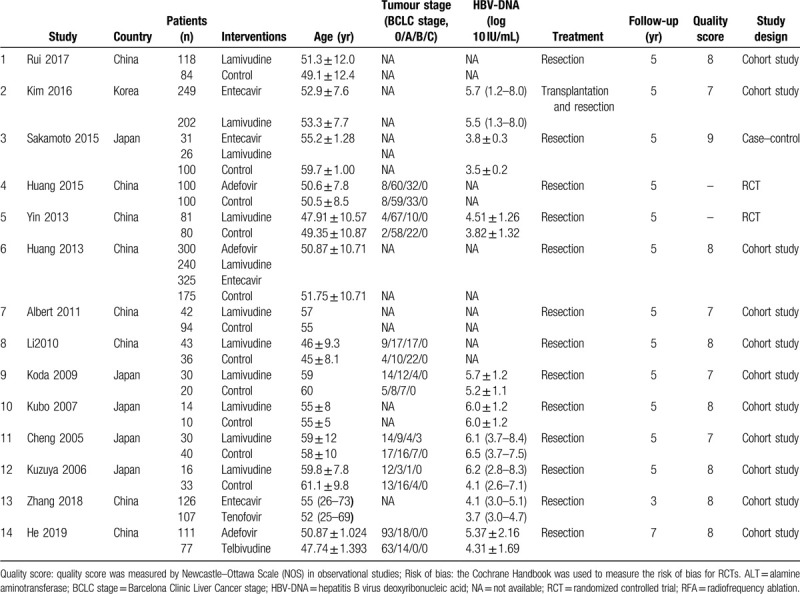
Patients’ characteristics in the included study.

### 3.2 Different effects of nucleos(t)ide analogues for HBV-related HCC after curative resection in overall survivals

**Figure 2:**
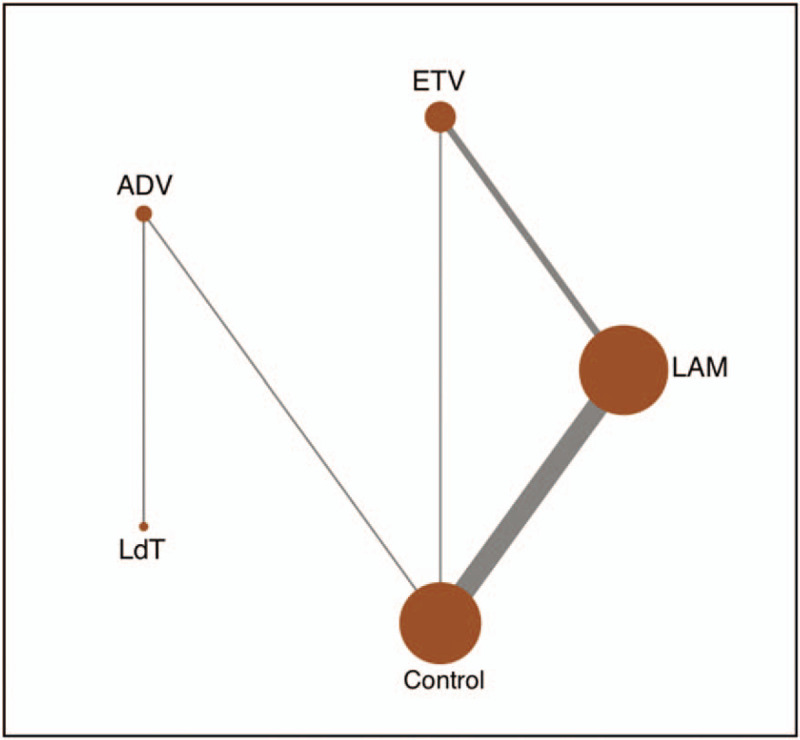
Network plot of different antiviral treatments. ADV, Adefovir dipivoxil; ETV, Entecavir; LAM, Lamivudine; LdT, Telbivudine.

Network connections of included trials were presented in Figure 2. This analysis includes 4 oral drugs for antiviral therapies, namely LAM, ETV, ADV, and LdT. It can be seen that LAM and ETV are the most studied treatments. As for ADV and LdT, despite the fact that it was included in 3 comparisons, its sample size was relatively large. Supplementary Figure 2 summarizes the contribution of direct comparisons in determining the network meta-analysis estimates for mixed and indirect evidence.

Table 2 summarizes the results of the network meta-analysis for 1-, 3-, and 5-year overall survivals. In 1-year overall survival, patients with nucleos(t)ide analogues as postoperative antiviral therapy has no significantly improved overall survival benefit at 1-year follow-up compared to the patients who did not. Significant improvement of 3-year overall survival was found in the treatment of ETV (OR: 2.41, 95% CI: 1.55–3.73) versus control group. For 5-year overall survival, ADV (OR: 2.35, 95% CI: 1.17–4.73), LAM (OR: 2.08, 95% CI: 1.78–5.58), and ETV (OR: 2.14, 95% CI: 1.59–2.88) were found to be more beneficial than control group.

**Table 2:**
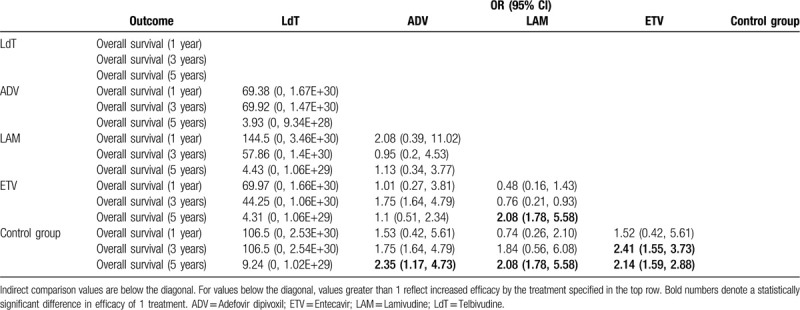
Results of network meta-analysis for 1-, 3-, 5-year overall survival rate of 4 nucleos (t)ide analogue treatments for HBV-related HCC patients after curative liver resection.

The induction treatment relative ranking of estimated cumulative probabilities of nucleos(t)ide analogues is demonstrated in Supplementary Figure 4. The SUCRA value rankings of overall survival rate for 1 year are ETV, ADV, LdT, control group, and LAM (SCURA scores are 71.2, 62.5, 55.5, 35.5, and 25.4, respectively), the SUCRA value rankings of overall survival rate for 3 years are ETV, LdT, LAM, ADV, and control group (SCURA scores are 70.8, 54.8, 53.9, 51.9, and 18.6, respectively), and for 5-year overall survival rate, rankings are ADV, ETV, LdT, LAM, and control group (SCURA scores are 66.3, 60.1, 57.8, 52.1, and 13.8, respectively) (Table 3). There is no significant publication bias from funnel plot in this network analysis (Supplementary Fig. 3).

**Table 3:**
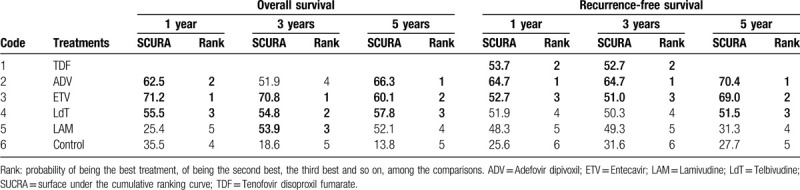
Ranking: probability from SUCRA of mean volume change, symptom score, and cosmetic score.

### 3.3 Different effects of nucleos(t)ide analogues for HBV-related HCC in recurrence-free survivals

**Figure 3:**
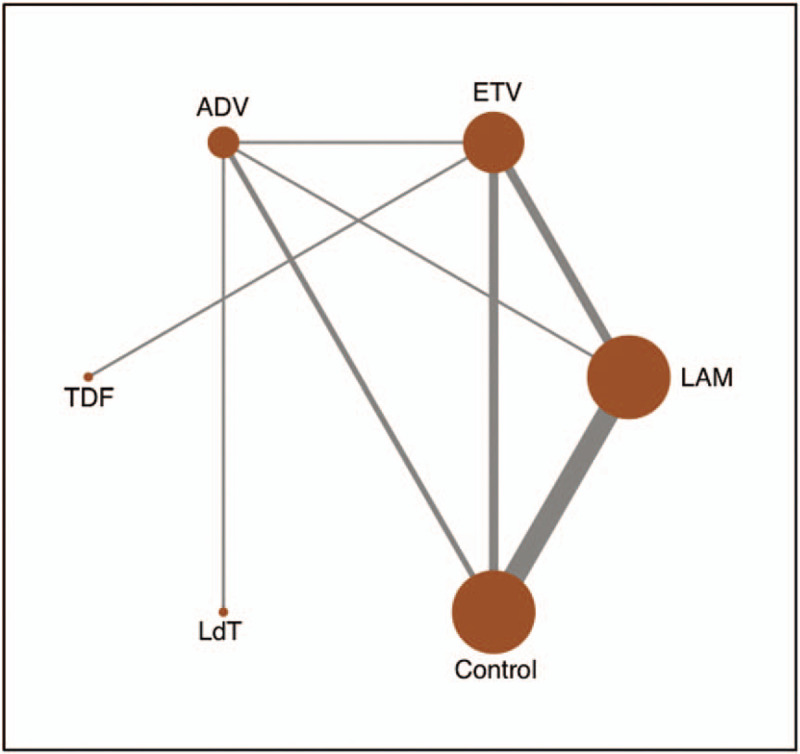
Network plot of different antiviral treatments. ADV, Adefovir dipivoxil; ETV, Entecavir; LAM, Lamivudine; LdT, Telbivudine; TDF, Tenofovir disoproxil fumarate.

The evidence-based network is presented in Figure 3. This analysis includes 5 nucleos(t)ide analogues for antiviral therapies, namely LAM, ETV, ADV, TDF, and LdT. Studies between LAM and control group are the most studied treatments. Supplementary Figure 5 summarizes the contribution of direct comparisons in determining the network meta-analysis estimates for mixed and indirect evidence.

In the results of network meta-analysis among all kinds of nucleos(t)ide analogues (Table 4), patients with ADV (OR = 1.92, 95% CI: 1.03–3.58) as postoperative antiviral therapy have significantly improved recurrence-free survival benefit at 1 year follow-up compared to the patients who did not while no significant improvement was shown in other nucleos(t)ide analogues. As for 3-year recurrence-free survival, ADV (OR = 1.57, 95% CI: 1.04–3.32), ETV (OR = 1.23, 95% CI: 1.05–2.80), and LAM (OR = 1.73, 95% CI: 1.06–2.82) significantly improved 3-year survival compared to control group while ADV (OR = 1.31, 95% CI: 1.25–3.12) has better benefit than LAM. In terms of late recurrence-free survival, ADV (OR = 1.88, 95% CI: 1.77–4.60), ETV (OR = 1.96, 95% CI: 1.36–2.55), and LAM (OR = 1.73, 95% CI: 1.06–2.82) all had better significant prognosis than patients without antiviral therapy postoperatively. Furthermore, patients with ADV as postoperative antiviral therapy has significantly recurrence-free survival benefit at 5-year follow-up compared to those undertaking ETV (OR = 1.96, 95% CI: 1.52–7.38) and LAM (OR = 1.39, 95% CI: 1.09–3.01).

**Table 4:**
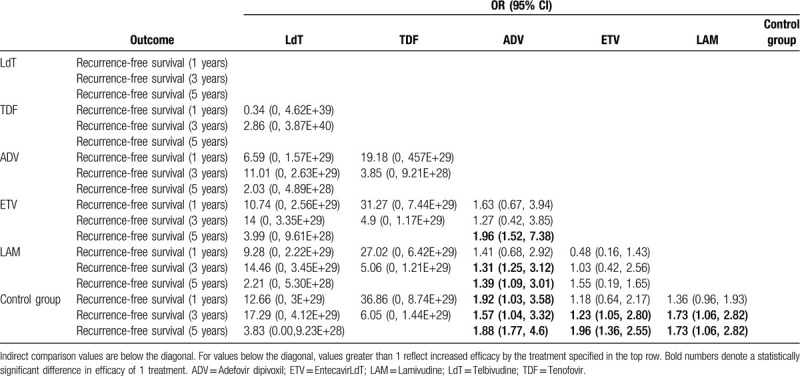
Results of network meta-analysis for 1-, 3-, 5-year recurrence-free survival of 5 nucleos(t)ide analogue treatments for HBV-related HCC patients after curative liver resection.

The induction treatment relative ranking of estimated cumulative probabilities of nucleos(t)ide analogues is demonstrated in Supplementary Figure 7. The SUCRA value rankings of recurrence-free survivals for 1 year are ADV, TDF, ETV, LdT, LAM, and control group (SCURA scores are 64.7, 53.7, 52.7, 51.9, 48.3, and 25.6, respectively), the SUCRA value rankings of recurrence-free survival for 3 years are ADV, TDF, ETV, LdT, LAM, and control group (SCURA scores are 64.7, 52.7, 51.0, 50.3, 49.3, and 31.6, respectively), and for 5-year recurrence-free survivals, rankings are ADV, ETV, LdT, LAM, and control group (SCURA scores are 70.4, 69.0, 51.5, 31.3, and 27.7, respectively) (Table 4). There is also no significant publication bias from funnel plot in the network analysis (Supplementary Fig. 6).

### 3.4 Quality assessment of trials and evidence grading

None of the eligible studies presented a severe risk of bias (Supplementary Fig. 1). Also, in the network meta-analysis, funnel plot analysis did not indicate any evident risk of publication bias (Supplementary Fig. 3 and 6). These findings, coupled with the absence of inconsistency and the lack of violation of the transitivity assumption, allowed to grade as high the strength of evidence advocating nucleos(t)ide analogues including ETV, TDF, ADV, LAM, and LdT better treatment than patients with no antiviral therapy after curative liver resection. All the other treatment comparisons were characterized by a confidence interval crossing the null value. Accordingly, their strength of evidence was graded as moderate.

## 4 Discussion

Due to there were a few RCTs about antiviral therapy for hepatitis B virus-related hepatocellular carcinoma after curative treatment, we adopted different methods to prevent potential bias. Ultimately, this study included observational studies with high quality sores. Besides, RCT methodological quality assessment shows these 2 RCTs (Gang Huang et al and Jianhua Yin et al)^[18,21]^ both have a low risk of bias mortality. The contribution plot demonstrates direct comparisons do not influence the entire network significantly (Gang Huang et al is below 10% in 1-, 3-, and 5-year recurrence-free survival).^[21]^ Moreover, the symmetrically distributed funnel plot indicates low risk of publication bias. In the single-agent induction treatments for hepatitis B, using antiviral therapies had a significantly better recurrence-free survival than none antiviral therapy, which also conforms to the previous evidence.^[16,17,19]^ In network meta-analysis, multiple treatment comparisons for a specific disease, which were not compared to each other, can be made simultaneously through a common comparator treatment. In our study, antiviral therapy showed a better prognosis than none antiviral therapy, but there is little differentiation between these 4 nucleos(t)ide analogues in overall survival. As for recurrence-free survival, ADV was more beneficial than ETV, LAM and control group. In Supplementary Figure 3 and 6, the SUCRA values provide the hierarchy for different antiviral treatments. For recurrence-free survival rate, ADV was observed with the highest ranking in 1-, 3-, and 5-year recurrence-free survivals with SUCRA values of 64.7%, 64.7%, and 70.4%, respectively.

In 2015, the American Association for the Study of Liver (AASLD) adopted ETV and TDF as first-line treatment for hepatitis B.^[44]^ However, given the necessity of long-term treatment in most patients with CHB, the importance of comparative data on the effectiveness and safety of nucleos(t)ide analogues is immense. In recent study, Murata and colleagues revealed that nucleotides, but not nucleosides, had the novel additional pharmacological effect of inducing IFN-λ3,^[45]^ which has potent efficacy on enhancement of HBV suppression. Furthermore, IFN-λ3 also showed antitumor activity in murine models of cancer, including hepatoma.^[46]^ Our results also found that ADV can obtain good outcomes in 1-, 3-, 5-year recurrence-free survival (64.7%, 64.7%, 70.4%), which is consistent with previous studies. As for the nucleoside analogues in the analysis (ETV, LdT, and LAM), ETV ranks higher than LAM, this probably due to the emergence of LAM-resistant HBV mutants. In the reported studies,^[47,48]^ the rate of LAM resistance was about 23% in 1 year to 71% in 5 years.

Collectively, although nucleos(t)ide analogues showed a promising future to be an alternative to the current first-line treatment, more RCTs are required to confirm this suggestion based on the following meta-analysis results:

(i) In the single-agent induction treatments for hepatitis B, using antiviral therapies had a significantly better survival benefit than none antiviral therapy.
(ii) Nucleotide analogues like ADV and TDF were proved to have superior benefit for the prognosis of recurrence-free survival for HBV-related solitary HCC patients after curative liver resection.

However, some limitations of this network meta-analysis should be discussed. First of all, most of the included studies are observational studies,^[9–11,14,22,39–43,49]^ So the results must be interpreted with caution. Second, some extent of heterogeneity in direct comparisons existed. In this study, we conducted the pooled estimate neglecting some of these factors. Third, there are a few RCTs or observational studies concerned the TDF antiviral therapy after curative treatment of hepatitis B virus-related hepatocellular carcinoma. As known in recent study,^[50–52]^ TDF are considered as the first-line treatment for hepatitis B. So more RCTs are required to conform this nucleotide analogue. Fourth, authors of some trials^[9,10,41–43]^ neglected to provide lucid and complete descriptions of critical information on methodology and findings, making it difficult to assess the extract data for meta-analysis.

## 5 Conclusions

This network meta-analysis indicates that patients with postoperative nucleos(t)ide analogues antiviral therapy had better survival benefit than those without antiviral therapy for HBV-related HCC patients after the curative treatment. Additionally, nucleotide analogues postoperative therapy like ADV and TDF has better impact on early and late recurrence-free survival of patients after curative treatment than those undertaking nucleoside analogues. While TDF has been listed as first-line treatment for hepatitis B, it might also have considerable clinical implications for preventing the recurrence of HCC in patients with CHB after curative treatment. Further studies for the efficacy of TDF to the prognosis of HBV-related HCC patients are needed to ensure the replicability of our findings.

## Acknowledgments

We would like to acknowledge Qiuping Zhang for editing the manuscript.

## Author contributions

## Supplementary Material

Supplemental Digital Content

## Supplementary Material

Supplemental Digital Content

## Supplementary Material

Supplemental Digital Content

## Supplementary Material

Supplemental Digital Content

## Supplementary Material

Supplemental Digital Content

## Supplementary Material

Supplemental Digital Content

## Supplementary Material

Supplemental Digital Content
