# Supplementary material for: The comparison of different antiviral therapies on the prognosis of hepatitis B virus-related hepatocellular carcinoma after curative treatments: A network meta-analysis
Source: Medicine (Baltimore). 2020 Aug 14;99(33):e20877. doi: 10.1097/MD.0000000000020877 (PMC7437757; doi:10.1097/MD.0000000000020877)
Supplement: Supplemental Digital Content [file medi-99-e20877-s002.docx]

The comparison of different antiviral therapies on the prognosis of hepatitis B virus-related hepatocellular carcinoma after curative treatments : a network meta-analysis. Zijing Xia
